# Revealing the mechanism of natural product-induced immunogenic cell death: opening a new chapter in tumor immunotherapy

**DOI:** 10.3389/fimmu.2024.1470071

**Published:** 2024-10-09

**Authors:** Yukun Chen, Zhenzhi Wang, Chi Zhang, Yisa Su, Tian Zhou, Kaiwen Hu

**Affiliations:** ^1^ Department of Oncology, Dong Fang Hospital, Beijing University of Chinese Medicine, Beijing, China; ^2^ Acupuncture and Tuina School, Chengdu University of Traditional Chinese Medicine, Chengdu, China

**Keywords:** natural products, ICD, tumor immunotherapy, tumor, cancer

## Abstract

This review underscores the role of natural products in inducing immunogenic cell death (ICD) as a key strategy in tumor immunotherapy. It reveals that natural products can activate ICD through multiple pathways—apoptosis, autophagy, pyroptosis, and necroptosis—leading to the release of danger-associated molecular patterns (DAMPs), dendritic cell activation, and improved antigen presentation, which together stimulate a potent anti-tumor immune response. The study also demonstrates the enhanced therapeutic potential of combining natural products with immune checkpoint inhibitors. With a focus on translating preclinical findings into clinical practice, this review consolidates recent discoveries and suggests future research paths, offering both theoretical insights and practical guidance for advancing cancer immunotherapy.

## 1 Introduction

**Figure 1:**
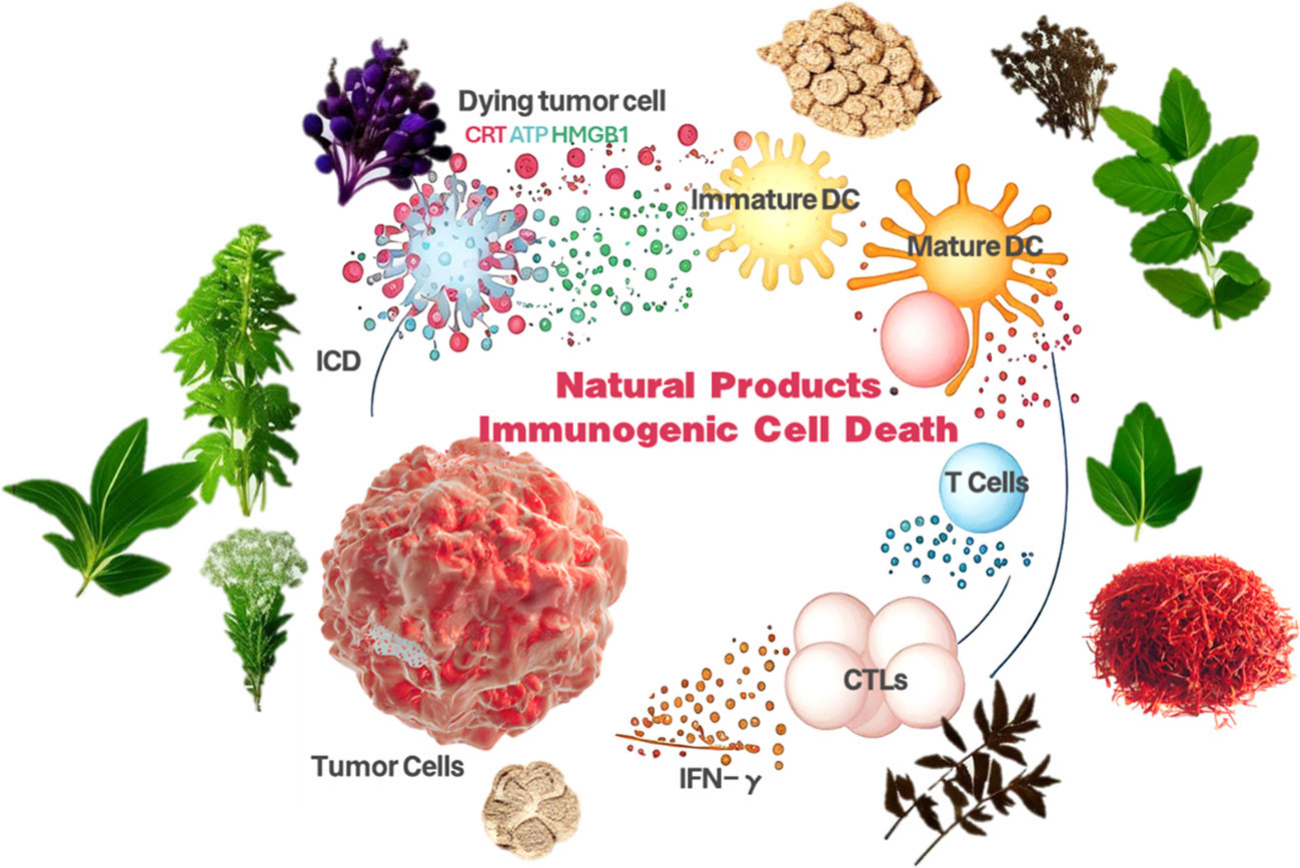
Natural product-induced immunogenic cell death schematic.

Cancer remains a significant global health concern, with millions of lives lost annually, as reported by the World Health Organization (WHO). Traditional treatments such as surgery, radiotherapy, and chemotherapy, while capable of managing tumors to some degree, are fraught with severe side effects and limited effectiveness, particularly in advanced stages (1). These treatments are also susceptible to recurrence and metastasis and can cause substantial damage to healthy tissues, compromising patients’ quality of life. Therefore, the quest for novel and effective cancer therapies is paramount. The tumor microenvironment, comprising tumor cells, stromal cells, blood vessels, and immune cells, is characterized by complex interactions that confer tumor adaptability and resistance to drugs (2). Immunosuppressive cells and molecules within this environment also undermine the immune system’s anti-tumor capabilities. Tumor immunotherapy represents a departure from conventional approaches by harnessing and amplifying the body’s immune response to target and eliminate cancer cells (3). The roots of tumor immunotherapy extend back to the 19th century, but substantial progress has been made in recent decades. Advances in understanding the immune system and tumor biology, coupled with the swift development of biotechnology, have elevated immunotherapy to a pivotal branch of cancer treatment. Notable approaches include Checkpoint Inhibitors: These, such as PD-1/PD-L1 and CTLA-4 inhibitors, work by disrupting immunosuppressive signals and reinvigorating T-cell activity, leading to a robust immune response against tumors (4). They have shown remarkable success in various cancers, including melanoma and lung cancer. CAR-T Cell Therapy involves genetically modifying a patient’s T-cells to target and eliminate cancer cells, with notable efficacy in hematologic cancers like acute lymphoblastic leukemia (5). Tumor Vaccines: These stimulate the body’s immune response by introducing specific tumor antigens to prevent or treat cancer. Numerous tumor vaccines are in clinical trials, demonstrating promising potential (6). Monoclonal Antibody Therapy: This targeted therapy acts against tumor-specific antigens by marking cancer cells for destruction or blocking growth signaling pathways (7). Immunotherapy has significantly improved patient survival, particularly in certain advanced cancers, outperforming traditional therapies regarding efficacy and long-term survival rates. It typically boasts lower toxicity and side effects, thereby enhancing the quality of life for patients, especially those with advanced stages of the disease (8). The individualized nature of immunotherapy is one of its key strengths; for instance, the effectiveness of PD-1/PD-L1 inhibitors is closely tied to PD-L1 expression levels in the tumor microenvironment (9). Combining immunotherapy with conventional treatments, such as pairing it with radiotherapy, can amplify the anti-tumor effect. However, some patients remain unresponsive or develop resistance to immunotherapy (10). Future research will focus on deepening our understanding of tumor immune evasion mechanisms, devising innovative therapeutic strategies, and identifying biomarkers that predict treatment response to refine treatment regimens and maximize efficacy. Immunotherapy, as an innovative strategy, has opened up a new path in the field of cancer treatment by stimulating and enhancing the immune system’s attack on tumors (11). ICD, as a key mechanism of cell death, provides a scientific basis and practical pathway for immunotherapy, significantly improving its efficacy by triggering the recognition and response of the immune system (12). Natural products, with their rich and diverse chemical structures and potential biological activities, are expected to become a crucial resource for inducing ICD and enhancing immunotherapy responses (13). These natural products may contain components that can initiate the ICD process or have the ability to modulate the immune system, thereby playing a supportive or synergistic role in immunotherapy (14). This article systematically elaborates on the induction pathways and mechanisms of action of ICD, aiming to provide scientific guidance for the efficient integration of natural products into immunotherapy strategies (15). It is expected to lay a theoretical foundation for future research and the development of more effective and safer cancer treatment methods. Through this in-depth exploration, we not only offer a new perspective for cancer treatment but also outline a clear blueprint for the application of natural products in tumor immunotherapy (16) (**Figure 1**).

## 2 Characterization of ICD

ICD is characterized by its unique immune activation mechanism: first, during ICD, cells release specific DAMPs, including calreticulin, high mobility group protein B1 (HMGB1), and heat shock proteins (HSPs), which are efficiently recognized by antigen-presenting cells of the immune system, such as dendritic cells (DCs), effectively recognize them (17). Second, ICD is closely associated with specific modes of cell death, although not all modes of apoptosis, necrotic apoptosis, autophagy, and iron death can trigger ICD. In addition, ICD activates cytotoxic T-cells (CTLs) and helper T-cells through the cross-presentation of DAMPs and tumor-associated antigens, thereby triggering a specific immune response against tumors (18). ICD can also stimulate a systemic immune response, which is not limited to local but systemic anti-tumor immune effects, which provides an important value for its application in tumor immunotherapy (19). Notably, the immune response triggered by ICD is highly tumor-specific, mainly targeting tumor cells and reducing the damage to normal tissues (20). Finally, ICD promotes the production of memory T cells, which can respond rapidly in the event of tumor recurrence and provide long-term anti-tumor immune protection for patients (21).

Biological markers of ICD include a series of critical molecules and structures that constitute an immune recognition signal for ICD. First, calreticulin translocates from the intracellular to the surface during ICD and acts as an “eat-me” signal to promote phagocytosis of tumor cells by antigen-presenting cells. Secondly, HMGB1 is released into the extracellular environment during cell death and binds to Toll-like receptors (TLRs), activating the immune response (22). In addition, ATP released during cell death acts as a signaling molecule that attracts surrounding immune cells, including DCs and macrophages, to participate in the immune response (23). Finally, the exosomes formed during cell death contain tumor-associated antigens, which can be captured by antigen-presenting cells and presented to T cells, further contributing to the activation of the immune system. These biological markers ensure that the ICD can effectively initiate an anti-tumor immune response (24).

ICD is induced by diverse pathways covering a wide range of therapeutic and biological mechanisms. The following are the main induction pathways of ICD and their applications in scientific research: Chemotherapeutic agents: Specific chemotherapeutic agents, such as anthracyclines and platinum-based drugs, not only reduce tumor burden by inducing tumor cell death but also enhance therapeutic efficacy by activating the immune system mechanisms (25). These drugs activate the immune response by inducing cell death while promoting the release of DAMPs. Radiation therapy: By inducing DNA damage and cell death, radiation therapy can trigger the release of DAMPs, which in turn activates the immune system (26). This combination of therapeutic modalities can kill tumor cells directly and enhance immune surveillance and clearance via the ICD pathway. Microbial Infections: Certain bacterial and viral infections can trigger an immune response by activating the ICD pathway (27). These microbial infections cause direct cellular damage and activate the immune system by mimicking cell death signals. Cellular Stress Response: Cells may respond to internal and external environmental stresses, such as oxidative stress and endoplasmic reticulum stress, through the ICD pathway (28). These stress responses lead to cell death and release immune-activating signals by modulating intracellular signaling pathways (29). Targeted therapy: Targeted therapeutic agents, such as immune checkpoint inhibitors, can promote ICD by deregulating the immune system from tumor cells. This therapeutic strategy not only targets specific molecular targets of tumor cells but also can remodel the tumor microenvironment and enhance the anti-tumor activity of immune cells (30). The discovery and in-depth study of these inducible pathways have provided new strategies and ideas for tumor therapy. Although the pathways mentioned above for inducing ICD show some potential in tumor therapy, they still have some limitations in practical application. Severe side effects may accompany chemotherapy and radiotherapy, and not all tumor types are sensitive to such treatments (31). Microbial infection-induced ICD may be challenging to control and presents safety concerns. Pathways for induction of cellular stress response may have variable effects depending on cell type and tumor microenvironment (32). Targeted therapies, although precise, may face problems with drug resistance and therapeutic response rate. Therefore, finding safer, effective, and widely applicable ICD induction methods is an important research direction in tumor therapy (33). In this context, natural products are ideal candidates for ICD induction due to their diverse biological activities, low toxicity, and potential immunomodulatory abilities, which deserve in-depth research and development (34).

## 3 Progress in the study of natural product-induced ICD

With their rich diversity and remarkable biological activities, natural products have become an essential focus in induced ICD research. These nature-derived compounds are diverse and exhibit unique potential in antitumor therapy. The following is an overview of some of the more intensively studied natural products and the mechanisms by which they induce ICD (**Table 1**).

**Table 1:** Natural products that induce immunogenic cell death and their mechanisms of action.

Classify	Name	Source	Cancer species	Molecular mechanism	References
Triterpenoids	Ginsenoside Rg3	ginseng stems	Lung cancer, melanoma	Rrising in CRT and HSP expression	(35)
Flavonoids	Baicalein	Scutellaria baicalensis	Gastric tumors	Inducing CRT and membrane-bound protein A1 translocating to the cytoplasmic membrane and releasing HMGB1 and ATP.	(36)
Flavonoids	curcumin	Turmeric	Glioma	Activating the endoplasmic reticulum stress PERK-eIF2α and IRE1α XBP1 signaling pathways	(37)
Polyphenolic	Resveratrol	Grapes, red wine, peanuts and some berries	Ovarian cancer	Triggering CRT protein cell surface exposure, HMGB1 secretion, and ATP release.	(38)
Terpenoids	Wogonin	Root of Scutellaria baicalensis Georgi	Gastric tumors, Melanoma	ROS production induces endoplasmic reticulum (ER) stress, including phosphorylation of PERK /PKR and eIF2α, activating PI3K/AKT and inducing CRT/membrane-associated protein A1 translocation.	(39)
Alkaloids	Camptothecin	Camptotheca acuminata	Breast cancer	Inhibiting topoisomerase I, triggering DNA double-strand breaks and damage	(40)
Alkaloids	Capsaicin	Peppers	Osteosarcoma	Inducing rapid translocation of CRT from the cell interior to the cell surface.	(41)
Other	Arsenic trioxide	Arsenic	Myeloma, bladder cancer	ATO inducing autophagy,apoptosis, iron death, and necrotic apoptosis pathways in cancer contributed to ATP release, HMGB1 version, CALR exposure of cancer cells, and IFNβ1 secretion of T cells.	(42)
Other	Psoralen	Fructus psoraleae	Breast cancer, colon cancer	Triggering a cellular stress response, including CRT outgrowth and HMGB1 release, and disrupting the degradative function of autophagic lysosomes and feedback-regulated the ERK1/2-mTOR p70S6K signaling pathway.	(43)
Other	Shikonin	Arnebia euchroma	Melanoma	Effectively promoting the expression of specific DAMPs, and triggering the cystatinase cascade reaction.	(44)

### 3.1 Triterpenoid

Triterpenoids are commonly found in plants and exhibit diverse biological activities. Ginsenoside Rg3 (Rg3) is a tetracyclic triterpenoid saponin isolated from ginseng stems, leaves or roots (45). In this study by Son et al., ginsenoside Rg3-mediated apoptotic tumor cell death and a consequent rise in calreticulin (CRT) and HSP expression on the surface of lung cancer (LLC) and melanoma cells (B16F10) were observed. Increased transcription of relevant apoptotic genes (e.g., BCL2L13, CALR, or HSP60) by mRNA microarray analysis further confirmed the Rg3-induced apoptosis of tumor cells and their increased surface protein expression. Gene transcription of HMGB1, another marker of ICD, was also significantly increased in Rg3-treated LLC and B16F10 tumor cells (2- to 4-fold) compared to controls. The property of CRT as an “eat-me” signal was confirmed by co-culture of Rg3-treated tumor cells with DCs. The property of CRT as an “eat-me” signal was confirmed by the co-culture of Rg3-treated tumor cells with DCs, as evidenced by the increase in the ratio of double-positive cells for both CD11c (a DC marker) and CRT detected by flow cytometry. A striking phenomenon in the study of Son et al. is that the Rg3-treated tumor cells secreted IFN-γ, which, as an anti-tumor effector molecule, is usually produced by T cells rather than by tumor cells. In addition, overexpression of IFN-γ in B16F10 cells was found to inhibit tumor growth and induce apoptosis. In addition to the inhibition of pro-angiogenic (TNF-α) and immunosuppressive cytokine (TGF-β) secretion by Rg3, the production of IFN-γ by Rg3-treated tumor cells suggests that Rg3 may be a promising strategy for anti-cancer immunotherapy. Although Rg3 was able to induce ICD in LLC (Lewis Lung Cancer) and B16F10 melanoma cells, various parameters of cell death, including surface molecules including CRT expression and cytokine secretion, exhibited different modulation patterns in these two cell lines. B16F10 melanoma cells were susceptible to Rg3-induced cell death due to their immunogenicity. However, B16F10 cells preferred necrosis-like cell death in the presence of Rg3 compared to LLC. Although the surface expression of CRT was similar in both tumor cell lines, HSPs were more prominently expressed in LLC, a typical non-immunogenic tumor cell. Also, a higher percentage of Rg3-treated melanoma cells were uptaken by DCs compared to lung cancer cells. The data of Son et al. demonstrated that Rg3 not only induces ICD in immunogenic tumors such as melanoma but also exerts the same effect in non-immunogenic tumors such as lung cancer cells. This property of Rg3 makes it ideal for use as an immunotherapeutic module. Crucially, Rg3-induced cell death can induce the transformation of non-immunogenic tumor cells into immunogenic tumor cells, thus enabling usually difficult-to-manage non-immunogenic tumor cells (e.g., lung cancer cells) to be sensitized to tumor immune responses (e.g., DC uptake) (35).

### 3.2 Flavonoids

Flavonoids are widely found in fruits and vegetables and possess various biological activities, including antioxidant and anti-inflammatory properties. Baicalein is a flavonoid extracted from the root of the traditional Chinese medicine *Scutellaria baicalensis*, which has a variety of biological activities (46). Yang et al. demonstrated for the first time that baicalein could trigger effective anti-tumor immunity by inducing CRT and membrane-bound protein A1 translocating to the cytoplasmic membrane and releasing HMGB1 and ATP. The study also revealed the signaling pathways involved in this process. We found that baicalein-induced reactive oxygen species (ROS) production triggered endoplasmic reticulum (ER) stress responses, including phosphorylation of PERK (PKR-like endoplasmic reticulum kinase) and eIF2α (eukaryotic initiation factor 2α), and that these phosphorylation events acted as upstream signals for the activation of phosphatidylinositol 3-kinase (PI3K)/AKT, which, in turn, induced CRT and membrane-associated protein A1 P22/CHP, a calcium 2+ -binding protein, is associated with CRT and is required for CRT translocation to the cell membrane. HMGB1 and ATP released from baicalein-treated MFC cells, alone or in combination with other potential factors, were able to activate DCs and induce cytokine release. *In vivo* studies further demonstrated that vaccination immunization with tumor cells pretreated with baicalein significantly inhibited the growth of homografted gastric tumors in mice and may have triggered an inflammatory response. In summary, ER stress-induced PI3K pathway activated CRT/Annexin A1 translocation (“eat me” signaling) and HMGB1 release, which mediated baicalein-induced tumor cell vaccination immunity. This suggests that baicalein is a novel and compelling candidate for immunotherapy against gastric tumors (36). Xiu et al. investigators explored the effects of curcumin on ionizing radiation-induced ICD in glioma cells under normoxic and hypoxic conditions and its mechanism of action *in vitro* and *in vivo*. Apoptosis and cell surface CRT exposure in hypoxic or normoxic glioma cells were examined by flow cytometry. Extracellular ATP and HSP70 were measured by chemiluminescence and ELISA, respectively. Endoplasmic reticulum (ER) stress protein levels were detected using Western blot. In addition, the induction of true ICD was detected by a vaccination test in mice carrying a glioma model. Splenic lymphocytes and tumor-infiltrating lymphocyte subpopulations were then analyzed by flow cytometry and immunohistochemistry. The results showed that curcumin pretreatment before X-ray irradiation significantly increased the rate of radiation-induced apoptosis in normoxic or hypoxic glioma cells. Curcumin enhanced radiation-induced CRT exposure, HSP70 and ATP release, and ER stress signaling pathway activity. Both apoptosis and CRT exposure induced by curcumin in combination with X-ray treatment were reduced when treated with an inhibitor of the ER stress pathway. In vaccination experiments, X-ray-irradiated glioma cells triggered a robust immunogenic response, resulting in the rejection of tumor formation in 70% of the mice. In contrast, cells treated with a combination of curcumin and X-rays triggered a more robust immune response, achieving rejection of tumor formation in 90% of mice. The combination treatment significantly increased the percentage of tumor-infiltrating CD4+, CD8+ T-lymphocytes, and CD11c+ Dcs compared to X-ray irradiation alone. Curcumin enhanced further glioma ICD induced by ionizing radiation under normoxic or hypoxic conditions by activating the endoplasmic reticulum stress PERK-eIF2α and IRE1α-XBP1 signaling pathways (37).

### 3.3 Polyphenols

**Figure 2:**
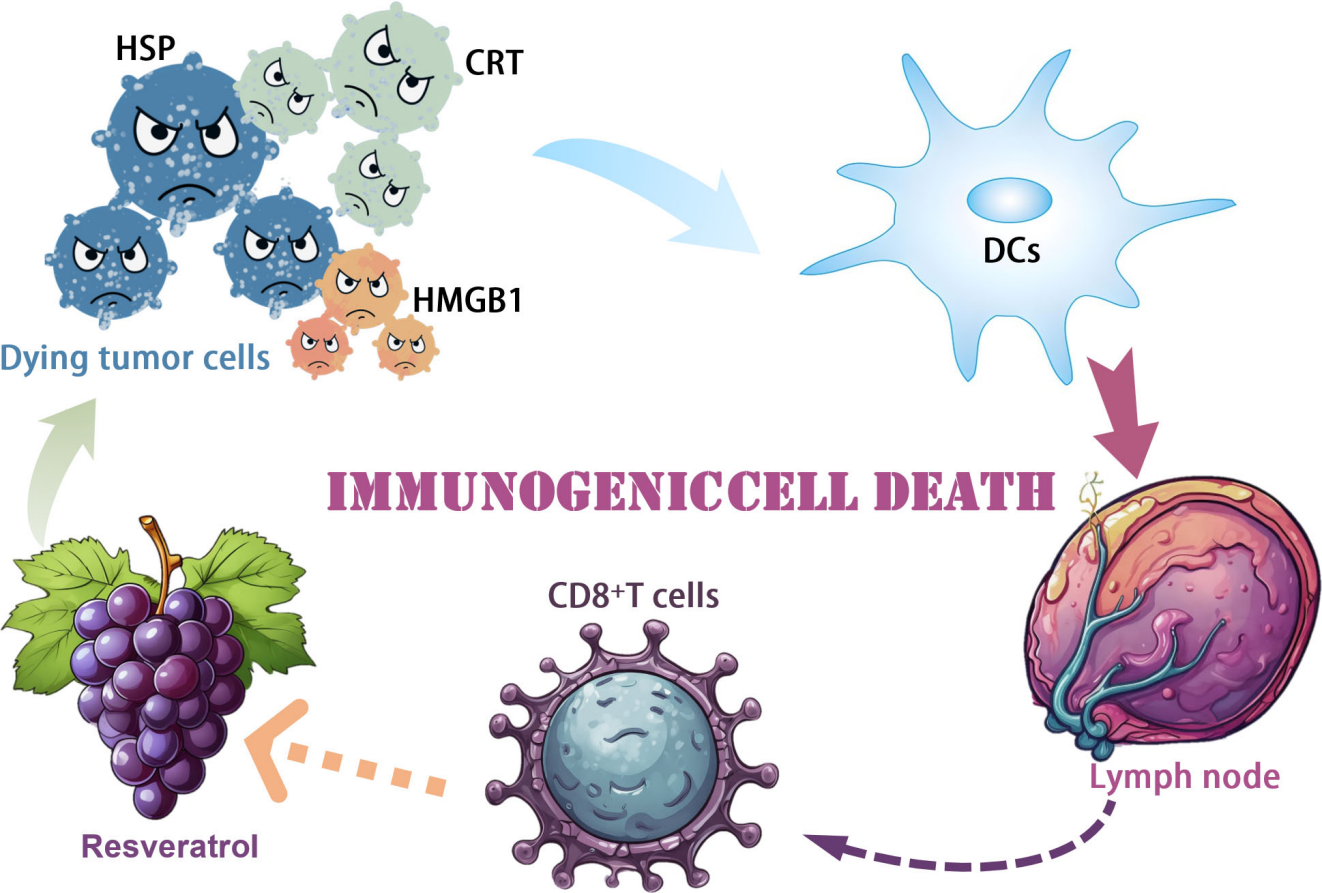
Mechanism of resveratrol induced immunogenic cell death.

Polyphenolic compounds have received widespread attention for their antioxidant and anticancer activities. Resveratrol is a naturally occurring polyphenol compound found primarily in grapes, red wine, peanuts and some berries. Given the recognized role of Resveratrol in immunomodulation in various diseases, it remains to be determined whether Resveratrol stimulates immune activation to promote tumor-killing effects (47). Zhang et al. demonstrated the antiproliferative and apoptosis-inducing effects of Resveratrol on ovarian cancer cells *in vitro*, which is consistent with previous reports. Zhang et al. demonstrated that RES treatment significantly triggered CRT protein cell surface exposure, HMGB1 secretion, and ATP release, suggesting the presence of potential ICD in this context, which is different from the typical apoptosis in clean, neat, and poor ICD processing. To test this hypothesis experimentally *in vivo*, we used the mouse ovarian cancer cell line ID8, pretreated with Resveratrol, and characterized for cell surface exposure to CRT. Vaccination with Resveratrol-pretreated ID8 significantly inhibited the growth of subsequently vaccinated xenograft tumors, demonstrating immune activation by Resveratrol treatment. The antitumor activity of Resveratrol was further shown in a subcutaneous mouse model of naïve ID8 cells, in which intraperitoneal administration of Resveratrol significantly inhibited tumor progression, accompanied by a decrease in cell proliferation and an increase in apoptosis. We further characterized the immune microenvironment in tumor tissues in response to Resveratrol treatment and found a significant increase in mature DCs and cytotoxic T cells. There was a substantial decrease in TGF-β secretion and a considerable decrease in IL12p7 and IFN-γ secretion. Most notably, the combination of the immune checkpoint inhibitor PD-1 antibody with Resveratrol significantly inhibited the growth of xenograft tumors. In contrast, the depletion of CD8+ cells partially restored the tumor progression affected by treatment with RES alone. Taken together, our data suggest that RES induces apoptosis and ICD in ovarian cancer *in vitro* and *in vivo*, which requires further mechanistic studies and clinical trials (38) (**Figure 2**).

### 3.4 Terpenoid

**Figure 3:**
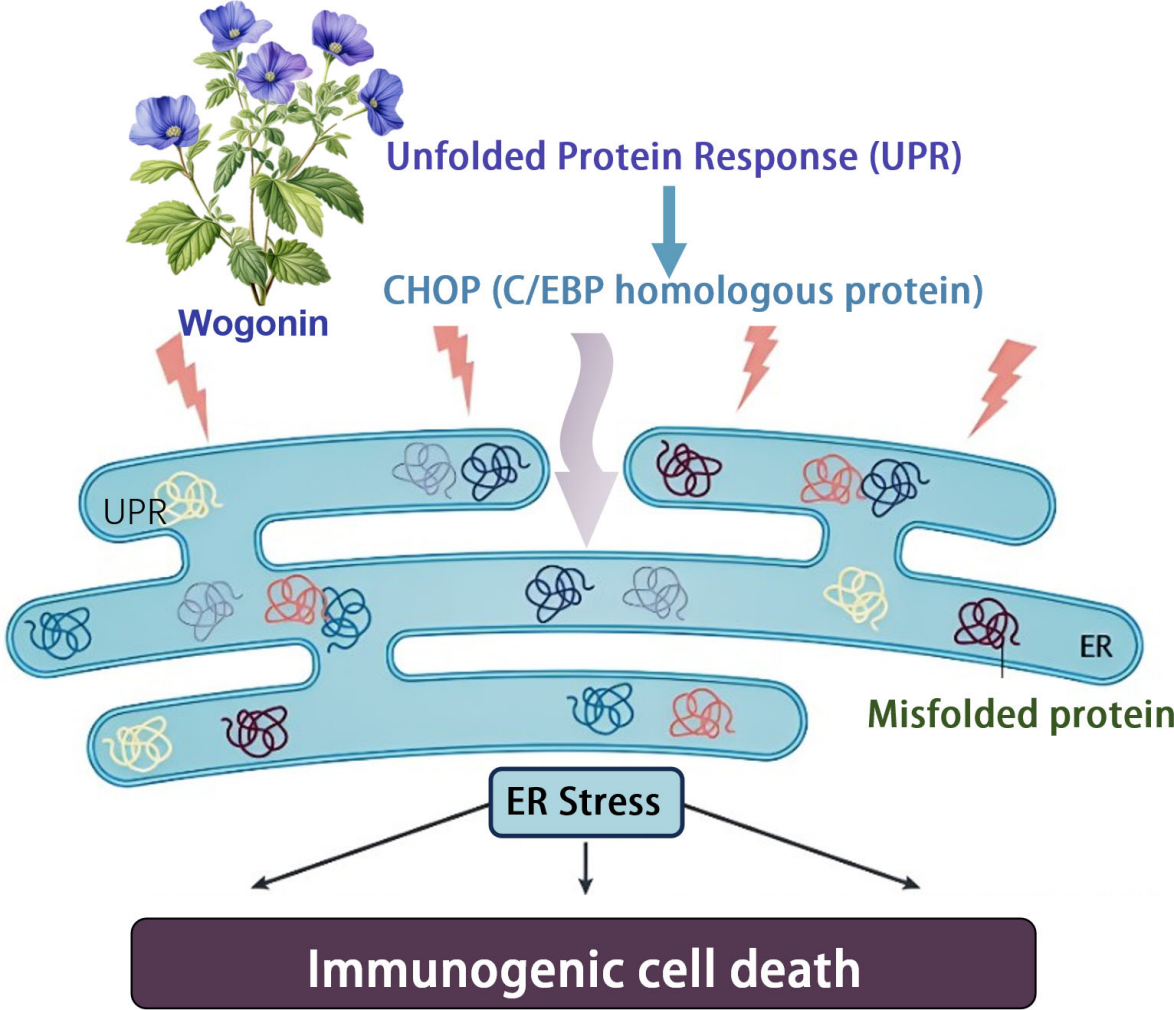
Baicalin induces immunogenic cell death through endoplasmic reticulum stress.

Terpenoids have a variety of pharmacological effects, including antitumor and anti-inflammatory effects. Wogonin is a kind of natural flavonoids, which mainly exists in the root of *Scutellaria baicalensis* Georgi. It is one of the main effective components in *Scutellaria baicalensis* (43). Wogonin has shown a variety of biological activities *in vivo* and *in vitro*, including anti-inflammatory, antioxidant, anticancer, antiviral, neuroprotective and other effects (48). Tripterine, a pentacyclic triterpenoid derived from *Tripterygium wilfordii*, has become the focus of oncology research due to its significant efficacy in immune response modulation and induction of immunogenic cell death (ICD). The specific properties of tripterine have recently been effectively used to develop innovative immunotherapy strategies, especially in the field of melanoma treatment research, which shows great potential application value. In a separate study, Qiu et al. demonstrated an immunotherapeutic strategy that does not require anti-PD-L1 antibodies - Celastrol nanoemulsion-based immunotherapy for melanoma. CELs in tumors effectively induced ICD, which activated DCs, recruited cytotoxic CD8+ T-cells and NK-cells, and increased the levels of immune-enhancing cytokines while decreasing immune-suppressing MDSCs and Tregs, resulting in potent systemic immunotherapy. More importantly, unlike other ICD inducers that increase PD-L1 expression in tumor cells and, therefore, must rely on more anti-PD-L1 monoclonal antibodies for their therapeutic effects, CEL simultaneously down-regulated PD-L1 expression in tumor cells, thereby directly activating recruited T cells. The developed nanoemulsion maintained a sustained tumor CEL concentration to induce ICD and PD-L1 downregulation effectively. As a result, low doses of CEL nanoemulsion effectively inhibited the growth of treated and untreated tumors and prolonged the survival of mice (39) (**Figure 3**).

### 3.5 Alkaloids

Camptothecin, a pale yellow needle-like crystalline plant anticancer component extracted from Camptotheca acuminata, has demonstrated significant antitumor potential. This component activates endoplasmic reticulum stress and mitochondrial pathways by inhibiting topoisomerase I, triggering DNA double-strand breaks and damage. This process leads to elevated intracellular Ca2^+^ concentrations and the production of reactive oxygen species (ROS), which trigger the expression of damage-associated molecular patterns (DAMPs) on the surface of tumor cells. Some studies have shown that in 4T1-Luc2 orthotopic tumor model, the anti-tumor effect of camptosome is significantly better than that of Onivyde. When used in combination with α-PD-L1 monoclonal antibody, it not only completely eliminated lung metastases, but also significantly prolonged the survival of mice. This combination therapy elicits a strong CTL response by increasing the expression of CD8 + T cells, CRT, HMGB-1, LRP-1, IFN-γ, and granzyme B infiltrating the tumor, effectively killing tumor cells (40).

### 3.6 Naphthoquinone

Shikonin is a kind of natural compound extracted from *Arnebia euchroma* and belongs to naphthoquinone compounds. It has a long history of use, mainly known for its anti-inflammatory, antibacterial, antiviral and antitumor effects (41). Chen et al. constructed a hypothetical model to elucidate the possible molecular pathways by which shikonin triggers ICD in non-immunogenic B16 tumor cells. Herein, it is briefly summarized: shikonin effectively promotes the expression of specific DAMPs, which triggers the cystatinase cascade reaction in therapeutic tumor cells. When combined with PAMPs, shikonin-triggered tumor cell lysates (TCLs) were able to comprehensively activate the phenotypic and functional maturation of DCs, which further facilitated the expansion of the Th17 cell population, particularly in terms of Th1 cell differentiation and cytotoxic T lymphocyte activation, which together effectively inhibited tumor growth and prolonged experimental survival of mice. In light of this, these findings suggest that shikonin has excellent potential for intensive development as an adjuvant for tumor cell lysate-loaded DC vaccines or other immunotherapeutic strategies (49).

### 3.7 Other natural products

Arsenic trioxide (ATO) has been recognized in traditional medicine for more than 2,000 years as a treatment for a variety of diseases, and has shown excellent efficacy especially in the treatment of acute promyelocytic leukemia (50). Chen et al. found that ATO is usually combined with all-trans retinoic acid (ATRA) to treat promyelocytic leukemia (PML) with considerable success. Studies have shown that ATRA is more effective in immunocompetent mice than in immunodeficient mice, but an immune-dependent effect of ATO has not been described in PML.ATO has been shown to potentiate the cytotoxicity of lymphokine-activated killing (LAK) on human myeloma cells to enhance the efficacy of BCG (Bacille Calmette-Guérin) vaccine in a mouse model of bladder cancer and also to deplete regulatory T cells. ATO triggers ICD in solid tumors, a process that involves apoptosis, necrotic apoptosis, pyroptosis, and iron death. ICD triggers the release of DAMPs, activates DCs, and facilitates tumor antigen presentation, stimulating an anti-tumor immune response. Chen et al. found that cancer cells treated with ATO could be used as a whole-cell vaccine and injected into mice to reduce cancer growth. This anticancer effect was dependent on CD8 T cells and interferon signaling. Mechanistically, ATO induces multiple cellular stress and death pathways, including autophagy, apoptosis, iron death, and necrotic apoptosis. Although these pathways are not essential individually, they significantly enhance ATO treatment’s anticancer effect. The authors noted that autophagy, apoptosis, iron death, and necrotic apoptosis pathways contributed significantly to ATO-induced ATP release, HMGB1 version, CALR exposure, and IFNβ1 secretion. Further studies revealed that whole-cell vaccination with ATO partially inhibited established tumor growth, an effect that required the expression of specific genes. In addition, combining the ATO vaccine with PD-1 blockade enhanced the ability to inhibit tumor growth. ATO may be the first antitumor chemotherapeutic agent to stimulate ICD, and its multi-pathway stress and death model may be more effective than a single or few pathways. These findings open new possibilities for developing more effective ICD-based therapeutic cancer vaccines (51). Alkaloids have significant antitumor activity. Capsaicin is an active ingredient found in chili peppers and belongs to alkaloids. It is irritating to mammals, including humans, and produces a burning sensation in the mouth. Capsaicin has been found to be useful as a cancer preventive agent and has shown widespread utility against various types of cancer (42). Jin et al. investigators delved into the role of capsaicin in inducing ICD. In this study, they evaluated the inhibitory effect of capsaicin and cisplatin on the growth of MG-63 cells using MTT assay. Through mitochondrial membrane potential assay and Western blot analysis, the researchers investigated the mechanism of apoptosis induced by capsaicin and cisplatin. They also examined the ability of capsaicin and cisplatin to induce CRT membrane translocation and mediate ICD in human osteosarcoma cells (MG-63). It was found that both capsaicin and cisplatin induced apoptosis in MG-63 cells, but notably, only capsaicin-induced rapid translocation of CRT from the cell interior to the cell surface. Capsaicin treatment of MG-63 cells significantly enhanced their phagocytosis by DCs, and these phagocytosed MG-63 cell DCs effectively stimulated IFN-γ secretion from lymphocytes. These results suggest that capsaicin, as an anticancer drug, has the potential to induce ICD production by human osteosarcoma cells *in vitro (* 52). Psoralen, a furanocoumarin compound found chiefly in the leguminous plant *Psoralea corylifolia*. Researchers have found that tonicin effectively inhibits the process of autophagy. Psoralen triggered a cellular stress response and induced ICD-associated features, including CRT outgrowth and HMGB1 release, in MDA-MB-231 and CT26 cancer cells. In addition, psoralens disrupted the degradative function of autophagic lysosomes and feedback-regulated the ERK1/2-mTOR-p70S6K signaling pathway. The complementing-induced ICD effect was validated by inoculating a mouse model with a vaccine prepared from complementing-treated CT26 cells and subsequently observing a rejection response against CT26 tumor cells. Impairment of autophagy function contributed to the complementin-induced ICD, as evidenced by the significant reduction of complementin-induced CRT exposure and HMGB1 release after Atg5 knockdown. Our study reveals that complementin acts as an innovative autophagy regulator and ICD inducer and has an undocumented role in autophagy in ICD. Thus, these findings emphasize the potential value of psoralen in cancer immunotherapy, suggesting its promising application as an ICD inducer and its potential to improve cancer therapy while reducing toxicity (44).

## 4 Outlook and discussion

Based on a comprehensive review of existing research, we can conclude that natural product-induced immunogenic cell death (ICD) has shown significant advantages in the field of cancer immunotherapy and has become an important part of clinical treatment strategies. ICD promotes tumor-specific immune response by activating the immune system and has potential synergistic effects when combined with existing immunotherapies, which can improve the therapeutic effect and benefit more patients. Natural product-induced ICDs also have lower toxicity and side effects, providing new treatment options for drug-resistant cancer patients. Future research should aim to shed light on the precise mechanisms of ICD, address the complexity of natural product compositions, and develop personalized treatment strategies based on individual differences. By optimizing treatment combinations, we aim to maximize efficacy while minimizing side effects, and to verify the safety and effectiveness of these approaches through rigorous clinical trials. The findings of this study highlight the important role of natural products in activating the tumor immune microenvironment, promoting apoptosis and other forms of cell death, primarily through ROS-induced endoplasmic reticulum stress responses and PERK/eIF2 α/ATF4 signaling pathways. These findings not only highlight the synergistic potential of natural products used in combination with other therapeutic drugs, but also suggest the possibility of nanomaterials in enhancing therapeutic effects. In order to promote the wide application of natural product-induced ICD in tumor therapy, it is urgent to further explore its clinical potential. At present, there is a lack of clinical research on relevant achievements. It is expected to significantly promote the development of tumor immunotherapy field and accelerate the progress of clinical trials by carrying out clinical trials as soon as possible and integrating these research achievements into clinical practice. Looking ahead, through continued research and clinical validation, we expect natural product-induced ICDs to become a standardized strategy in cancer treatment, providing patients with more effective and safer treatment options.
